# Introductions and early spread of SARS-CoV-2 in France, 24 January to 23 March 2020

**DOI:** 10.2807/1560-7917.ES.2020.25.26.2001200

**Published:** 2020-07-02

**Authors:** Fabiana Gámbaro, Sylvie Behillil, Artem Baidaliuk, Flora Donati, Mélanie Albert, Andreea Alexandru, Maud Vanpeene, Méline Bizard, Angela Brisebarre, Marion Barbet, Fawzi Derrar, Sylvie van der Werf, Vincent Enouf, Etienne Simon-Loriere

**Affiliations:** 1 Evolutionary genomics of RNA viruses, Institut Pasteur, Paris, France; 2 Université de Paris, Paris, France; 3 These authors contributed equally; 4 National Reference Center for Respiratory Viruses, Institut Pasteur, Paris, France; 5 Molecular Genetics of RNA Viruses, CNRS - UMR 3569, University of Paris, Institut Pasteur, Paris, France; 6 Mutualized Platform of Microbiology, Pasteur International Bioresources Network, Institut Pasteur, Paris, France; 7 National Influenza Centre, Viral Respiratory Laboratory, Algiers, Algeria; 8 These authors co-supervised this work

**Keywords:** SARS-CoV-2, phylogeny, virus genomics, outbreak surveillance

## Abstract

Following SARS-CoV-2 emergence in China, a specific surveillance was implemented in France. Phylogenetic analysis of sequences retrieved through this surveillance suggests that detected initial introductions, involving non-clade G viruses, did not seed local transmission. Nevertheless, identification of clade G variants subsequently circulating in the country, with the earliest from a patient who neither travelled to risk areas nor had contact with travellers, suggests that SARS-CoV-2 might have been present before the first recorded local cases.

Severe acute respiratory syndrome coronavirus 2 (SARS-CoV-2) was identified as the cause of an outbreak of severe respiratory infections in Wuhan, China in December 2019 [1]. Despite strict quarantine measures in Wuhan and surrounding areas, the virus, responsible for coronavirus disease (COVID-19), rapidly spread across the globe, leading the World Health Organization (WHO) to declare a pandemic on 11 March 2020. Soon after the emergence of the virus, a specific syndromic surveillance for COVID-19 was implemented in France. Because viral genomics, coupled with modern surveillance systems can help to understand outbreak dynamics [2], we sequenced SARS-CoV-2 genomes from clinical cases sampled through the surveillance. 

## Surveillance of COVID-19 in northern France

Strengthened surveillance of COVID-19 cases was implemented in France on 10 January 2020, with the objective of identifying imported cases early to prevent secondary transmission in the community. In this context, the first cases detected by the National Reference Center for Respiratory Viruses (NRC) hosted at Institut Pasteur, Paris, happened to be the first identified in Europe. As the COVID-19 epidemic progressed in the country, the task of identifying SARS-CoV-2 infections was shared with the NRC-associated laboratory in Lyon and then extended to first line hospital laboratories in the whole country, with the NRC at Institut Pasteur focusing on the northern part of France, including the densely populated capital. Screening and sampling for SARS-CoV-2 was targeted towards individuals who had symptoms (fever and/or respiratory problems) or a travel history to risk areas for infection [3]. As the virus continued to spread, it became clear that COVID-19 patients could exhibit greatly variable clinical characteristics [4], including a proportion presenting with asymptomatic infection or mild disease [5].

## Patient sampling and analysis of retrieved SARS-CoV-2 genomes

**Figure 1:**
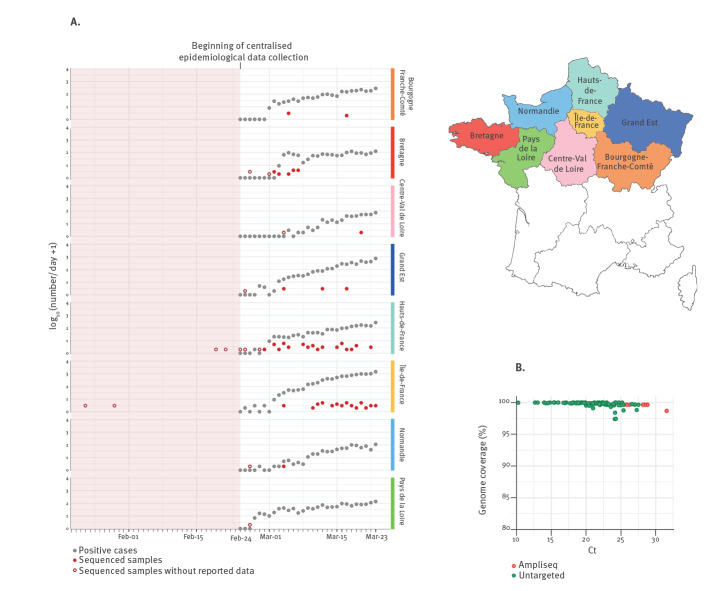
SARS-CoV-2 genome sequencing effort in northern French regions, 24 January–23 March 2020

We generated complete SARS-CoV-2 genome sequences from nasopharyngeal or sputum samples sent to the NRC at the Institut Pasteur as part of the ongoing surveillance (Figure 1A). We combined the SARS-CoV-2 genome sequences generated here, including 97 from northern France and three from Algeria with recent history of travel to France, with 338 sequences published and freely available from the Global Initiative on Sharing All Influenza Data (GISAID) EpiCoV database and/or GenBank. This dataset enabled to perform a phylogenetic analysis to gain more insight into the initial introductions and spread of the virus in France. More details on the methods used can be found in the Supplementary Material.

Ampliseq: amplicon-based sequencing; Ct: cycle threshold; number/day: number of laboratory-confirmed cases per day; SARS-CoV-2: severe acute respiratory syndrome coronavirus 2.

A. The plot represents the numbers of daily sequenced genomes in this study (red filled or hollow circles) overlaid with the number of reported positive cases (grey circles) obtained from Santé Publique France (www.santepubliquefrance.fr). Hollow circles indicate samples obtained on dates with zero reported SARS-CoV-2 positive cases. The data are shown separately for each region of northern France as indicated on the map on the right.

B. Percentage of SARS-CoV-2 genome coverage in relation to the Ct values obtained from the SARS-CoV-2 real-time reverse transcription PCR on the original samples, for the 97 genomes reported here. For reliability, amplicon-based sequencing was implemented for samples with Ct values higher than 25. Colours indicate sequencing approach: untargeted metagenomics (green) or amplicon-based sequencing (red).

## Ethical statement

Samples used in this study were collected as part of approved ongoing surveillance conducted by the NRC at Institut Pasteur (WHO reference laboratory providing confirmatory testing for COVID-19). The investigations were carried out in accordance with the General Data Protection Regulation (Regulation (EU) 2016/679 and Directive 95/46/EC) and the French data protection law (Law 78–17 on 06/01/1978 and Décret 2019–536 on 29/05/2019).

## Detected early viral introductions appear not to have seeded local transmission 

**Figure 2:**
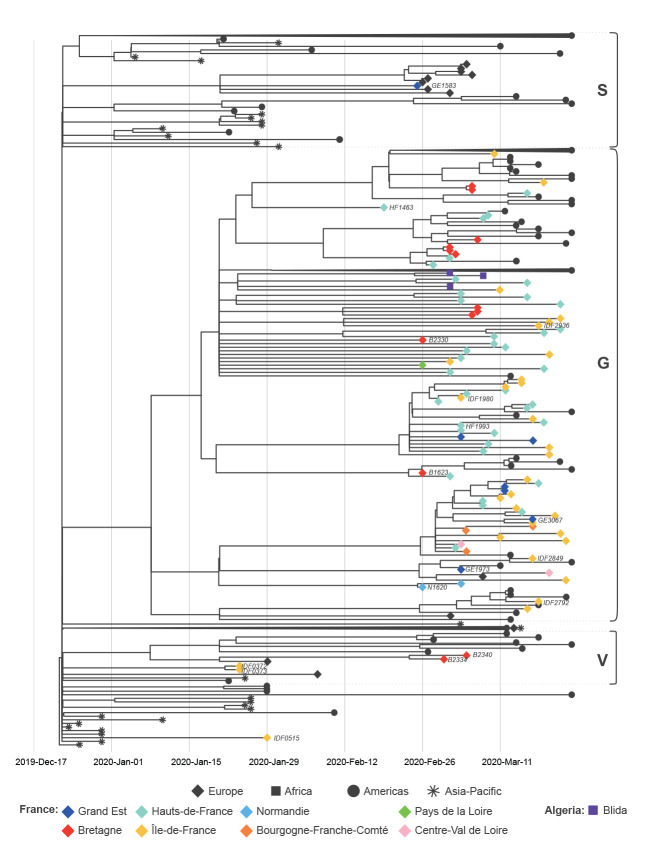
Phylogenetic analysis of sequences of early introductions and subsequently circulating SARS-CoV-2 in northern France, 24 January–23 March 2020

GISAID: Global Initiative on Sharing All Influenza Data; SARS-CoV-2: severe acute respiratory syndrome coronavirus 2.

**Figure 3:**
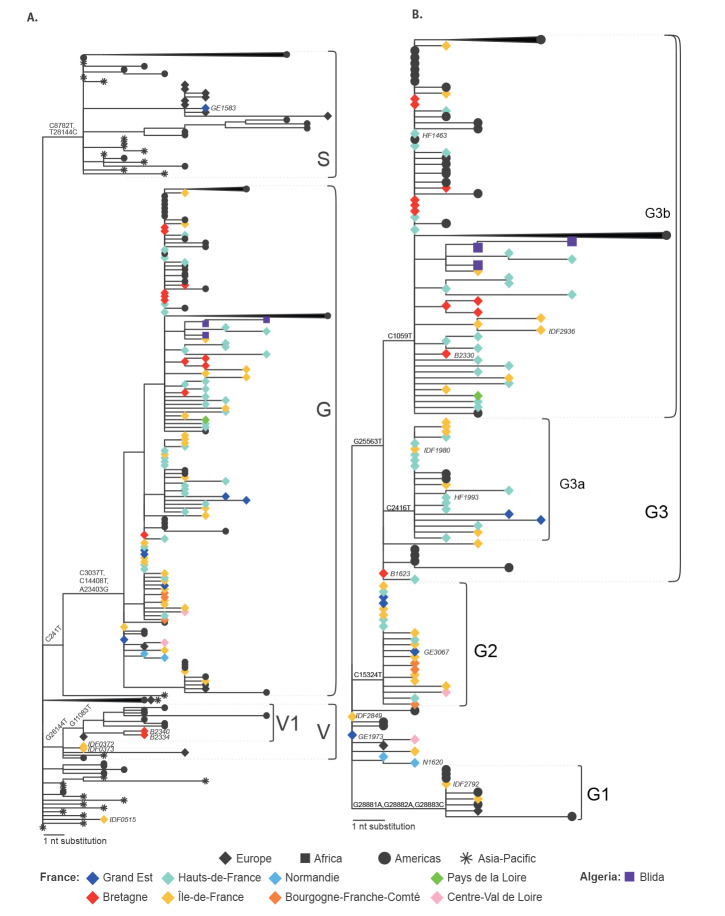
Phylogenetic trees with SARS-CoV-2 sequences showing (A) clades S,G,V and (B) clade G, with details on corresponding lineages, northern France, 24 January–23 March 2020

GISAID: Global Initiative on Sharing All Influenza Data; SARS-CoV-2: severe acute respiratory syndrome coronavirus 2.

Our analysis indicates that the quarantine imposed on the initial imported COVID-19 cases, who were captured by the syndromic surveillance in France, appears to have prevented local transmission. The first European cases, who were originally in Île-de-France (IDF) and who were previously described elsewhere [6], were direct imports from Hubei, China. They were sampled on 24 January 2020 and the two derived respective viral genomes, IDF0372 and IDF0373, fall accordingly near the base of the tree, within clade V, according to GISAID nomenclature (Figure 2, Figure 3A). Clade V is characterised by sequences with a T nucleotide at position 26144, instead of a G, corresponding to a V amino acid, rather than a G, at position 251 of non-structural protein 3a. The IDF0372 and IDF0373 genomes were identical and both harboured a G22661T non-synonymous mutation (V367F) in the receptor-binding domain of the spike protein, not observed in other genomes. Similarly, IDF0515, obtained from a 29 January sample, corresponds to a traveller from Hubei, China. This basal genome falls outside of the three major GISAID proposed clades V, G, and S (Figure 2), but carries the G11083T mutation associated with putative lineage V1 (Fig. S2), suggesting convergent evolution or a reversion of the V-clade defining G26144T change. Subsequent early cases detected in February in the West (Bretagne; B) or East (Grand Est; GE) of France (B2334/B2340, clade V and GE1583, clade S), all with recent history of travel to Italy, add to the genomic diversity of viruses from northern Italy, but also do not appear to have seeded local transmission within the current sampled sequence set (Figure 2).

Time calibrated tree of 438 SARS-CoV-2 sequences including northern France, Algeria and publicly available global sequences. The tree is rooted using the reference strain Wuhan/Hu-1/2019n (GenBank accession number: MN908947). The tips of the tree are shaped and coloured according to sampling location. Branch lengths are proportional to the time span from the sampling date to the inferred date of the most recent common ancestor. The three major clades according to GISAID nomenclature are indicated. Strain names of the sequences discussed in this study are indicated next to the corresponding tips.

The tips of the trees are shaped and coloured according to sampling location. Branch lengths are proportional to the number of nucleotide substitutions from the reference and tree root Wuhan/Hu-1/2019 (GenBank accession number: MN908947). GISAID clades and putative lineages are indicated on the right of each panel. Strain names of the sequences discussed in this study are indicated next to the corresponding tips in italic. Nucleotide substitutions shared among all the sequences of each clade or lineage are indicated next to the corresponding nodes. Some monophyletic lineages are collapsed for ease of representation. A complete tree is shown in Fig. S1.

## Clades and lineages of SARS-CoV-2 further circulating in northern France

All other sequences from northern France fall in clade G, defined by two synonymous mutations (C241T, C3037T) and one non-synonymous substitution (A23403G) corresponding to a D614G mutation in the spike protein (Figure 3), and this includes sequences captured during the steep increase of reported cases in many strongly affected regions (Figure 1). While a more thorough sampling will be needed to confirm this hypothesis, the phylogenetic analysis of sequences recovered in the current study suggests that the French outbreak was mainly seeded by one or several variants of this clade, unlike what is observed for many other European countries (https://nextstrain.org/ncov/europe?f_region=Europe) [7,8]. This clade can be further classified into lineages (putatively named G1, G2, G3, G3a, G3b), albeit supported again by only one to three substitutions. The lineages are for the most part respectively represented by sequences from several regions. Several genomes correspond to patients in GE, Normandie (N), IDF, Hauts-de-France (HF) and B with recent history of travel in Europe (GE3067, N1620, IDF2792), United Arab Emirates (IDF2936), Madagascar (HF1993), Egypt (B1623, B2330) or linked to Paris airports (IDF1980). These genomes might represent additional introductions of the same clade, since the respective cases tested positive for the virus when other local G-clade-virus infections had already been detected in the north of France. On the other hand, in lineage G3b, three sequences sampled in Algeria are closely related to sequences from northern France and likely represent exported cases in light of recent history of travel to France.

The syndromic surveillance allowed to capture one of the earliest representatives of clade G (HF1463, sampled on 19 February) (Figure 2). Importantly, this sequence carries two additional mutations compared with the reconstructed ancestral sequence of this clade (Figure 3B). Other sequences sampled weeks later (IDF2849, GE1973) are more basal to the clade, highlighting the complexity and risk of inferences based on 1 or 2 nucleotide substitutions. Because of this, and the scarcity of early sequences in many countries in Europe, country and within-country level phylogeographic estimations are unreliable with the current dataset. It is thus impossible to infer with confidence how the virus was introduced to France from the epicentre of the outbreak, and multiple routes are possible.

## Discussion

The generated genomes in this study provide more insight into the SARS-CoV-2 clades and variants circulating in northern France at the beginning of the outbreak and later during the pandemic. Results of the analyses seem to indicate that, at least for the first imported cases who could be captured by the surveillance, these introductions did not lead to further transmission of the virus in the community. Indeed, sequences from imported cases detected early in the outbreak did not belong to clade G, a clade identifying all the genomes retrieved later in the epidemic. Within clade G, a number of variants could be observed. Moreover, the earliest patient infected with a representative of clade G (HF1463) had no history of travel or contact with returning travellers, suggesting that the virus was silently circulating in France in February, a scenario compatible with the large proportion of persons with mild disease or asymptomatic infections [5], and observations in other European countries [9,10]. While this is also compatible with the time to the most recent common ancestor estimate for clade G (Figure 2), the current sampling clearly prevents reliable inference for the timing of introduction in France. Moreover while the current data may lead to hypothesize that the French outbreak could have been mainly seeded by one or several variants of the G clade, more data will be needed to confirm this. Another explanation would be that while the outbreak began with viruses belonging to various clades, the clade G might have become dominant in the north of France as the epidemic progressed.

Crucially, while all early symptomatic suspected COVID-19 cases samples were sent to the NRC for testing, this was no longer the case as the epidemic developed (Figure 1A). In addition, pauci- or asymptomatic cases are scarcely represented in our dataset. This study also reveals areas for potential improvement of SARS-CoV-2 genomic surveillance in France as several regions are poorly represented (Figure 1A). This is likely due to the heavy burden on hospitals, which while being able to perform local testing owing to the rapid sharing of molecular detection tools by the NRC, might have had to reduce the number of positive samples sent for confirmation and sequencing to the NRC. Because of this, and of the syndromic-only based surveillance, we likely underestimate the genetic diversity of SARS-CoV-2 circulating in France.

In conclusion, our study sheds light on the origin and diversity of the COVID-19 outbreak in France with insights for Europe, and highlights the challenges of containment measures when a significant proportion of cases are asymptomatic.

## Data and materials availability

The assembled SARS-CoV-2 genomes generated in this study were deposited on the GISAID database (https://www.gisaid.org/) as soon as they were generated, accession numbers can be found in Data S1 (Table S2).

## Supplementary Data

837000 Supplementary Material
