# Supplementary material for: Introductions and early spread of SARS-CoV-2 in France, 24 January to 23 March 2020
Source: Euro Surveill. 2020 Jul 2;25(26):2001200. doi: 10.2807/1560-7917.ES.2020.25.26.2001200 (PMC7346363; doi:10.2807/1560-7917.ES.2020.25.26.2001200)
Supplement: Supplementary Material [file 2001200_SimonLoriere_Supplementary_Material.pdf]

Supplementary Materials for

Introductions and early spread of SARS-CoV-2 in France, 24 January to 23 march 2020

"This supplementary material is hosted by *Eurosurveillance* as supporting information alongside the article *Introductions and early spread of SARS-CoV-2 in France, 24 January to 23 march 2020*, on behalf of the authors, who remain responsible for the accuracy and appropriateness of the content. The same standards for ethics, copyright, attributions and permissions as for the article apply. Supplements are not edited by *Eurosurveillance* and the journal is not responsible for the maintenance of any links or email addresses provided therein."

This file includes:

Materials and Methods
Figs. S1 to S2
Data S1 to S4
References

Materials and Methods

Sample collection

After reports of severe pneumonia in late December 2019, enhanced surveillance was implemented in France to detect suspected infections. For each suspected case, respiratory samples from the upper respiratory tract (nasopharyngeal swabs or aspirates) and when possible from the lower respiratory tract, were sent to the NRC, to perform SARS-CoV-2-specific real-time RT-qPCR. Demographic information, date of illness onset, and travel history were obtained when possible. A subset of samples were selected according to the viral load and their sampling location in order to have a broad representation across different regions of France.

Molecular test

RNA extraction was performed with the NucleoSpin Dx Virus Extraction kit (Macherey Nagel). RNA was extracted from 100 µl of specimen, eluted in 100 µl of water and used as a template for RT-qPCR. Samples were tested with a one-step RT-qPCR using three sets of primers as described on the WHO website (https://www.who.int/docs/default-source/coronaviruse/real-time-rt-pcr-assays-for-the-detection-of-sars-cov-2-institut-pasteur-paris.pdf?sfvrsn=3662fcb6_2).

Virus Sequencing

Viral genome sequences were generated by two different approaches. The first consisted in direct metagenomic sequencing, which resulted in complete or near complete genome sequences for samples with viral load higher than 1.45×10^4 viral genome copies/µl, which corresponds to a Ct value of 25.6 with the IP4 primer set (Fig. 1B, Data S1, Table S3).

Briefly, extracted RNA was first treated with Turbo DNase (Ambion) followed by purification using SPRI beads Agencourt RNA clean XP (Beckman Coulter). RNA was converted to double stranded cDNA. Libraries were then prepared using the Nextera XT DNA Library Prep Kit (Illumina) and sequenced on an Illumina NextSeq500 (2×150 cycles) on the Mutualized Platform for Microbiology (P2M) at Institut Pasteur.

For samples with lower viral load, we implemented a highly multiplexed PCR amplicon approach [1] using the ARTIC Network multiplex PCR primers set v1 (https://artic.network/ncov-2019), with modification as suggested in [2]. Synthesized cDNA was used as template and amplicons were generated using two pooled primer mixtures for 35 rounds of amplification. We prepared sequencing libraries using the NEBNext Ultra II DNA Library Prep Kit for Illumina (New England Biolabs) and barcoded with NEBNext Multiplex Oligos for Illumina (Dual Index Primers Set 1) (New England Biolabs). We sequenced prepared libraries on an Illumina MiSeq using MiSeq Reagent Kit v3 (2×300 cycles) at the biomics platform of Institut Pasteur.

Genome assembly

Raw reads were trimmed using Trimmomatic v0.36 [3] to remove Illumina adaptors and low quality reads, as well as primer sequences for samples sequenced with the amplicon-based approach. We assembled reads from all sequencing methods into genomes using Megahit, and also performed direct mapping against reference genome Wuhan/Hu-1/2019 (NCBI Nucleotide – NC_045512, GenBank – MN908947) using the CLC Genomics Suite v5.1.0 (QIAGEN). We then used SAMtools v1.3 to sort the aligned bam files and generate alignment statistics [4]. Aligned reads were manually inspected using Geneious prime v2020.1.2 (2020) (https://www.geneious.com/), and consensus

sequences were generated using a minimum of 3X read-depth coverage to make a base call. No genomic deletions were detected in the genomes analyzed.

Phylogenetic analysis

A set of 100 SARS-CoV-2 sequences generated in this study (97 from France, 3 from Algeria) was complemented with 338 genomes published or freely available sequences on GenBank or the GISAID database. From the latter, only published sequences were chosen [5, 6] (Data S1, Table S2). A total of 438 full genome sequences were analyzed with augur and auspice as implemented in the Nextstrain pipeline [7] version from March 20, 2020 (https://github.com/nextstrain/ncov). Within the pipeline, sequences were aligned to the reference Wuhan/Hu-1/2020 strain of SARS-CoV-2 (GenBank accession MN908947). The alignment was visually inspected and sequences from France were subset to analyze shared SNPs. No evidence of recombination was detected using RDP v4.97 [8]. A maximum likelihood phylogenetic tree was built based on the GTR model, after masking 130 and 50 nucleotides from the 5' and 3' ends of the alignment, respectively, as well as single nucleotides at positions 18529, 29849, 29851, 29853 to reduce the possibility of including variants due to assembly artifacts as performed in Fauver *et al.*, 2020 [5] and following the Nextstrain implementation for SARS-CoV-2. We checked for temporal signal using Tempest v1.5.3 [9]. The temporal phylogenetic analyses were performed with augur and TreeTime [10], assuming clock rate of 0.0008±0.0004 (SD) substitutions/site/year [11], coalescent skyline population growth model and the root set on the branch leading to the Wuhan/Hu-1/2020 sequence. The time and divergence trees were visualized with FigTree v1.4.4 (http://tree.bio.ed.ac.uk/software/figtree/). Nucleotide substitutions from the reference

sequence that define internal nodes of the tree were extracted from the final Nextstrain build file and annotated on the tree using a custom R script (https://www.R-project.org. Adobe Illustrator 2020 was used to prepare final tree figures. Sequence metadata (Data S1, Table S2), Nextstrain build, and R script is available at https://github.com/Simon-LoriereLab/SARS-CoV-2-France. The phylogeny can be visualized interactively at https://nextstrain.org/community/Simon-LoriereLab/SARS-CoV-2-France. In this study, we used the proposed nomenclature from GISAID to annotate three major clades V, G, and S according to specific single-nucleotide polymorphisms that are shared by all sequences in the clade. Clade defining variants according to GISAID nomenclature are included in Data S1, Table S1.

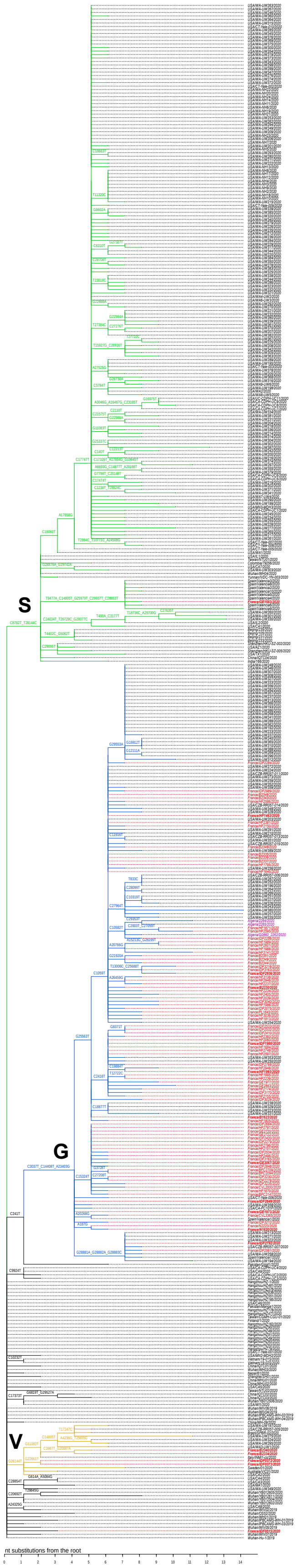
S
G
V
nt substitutions from the root

Fig. S1. Phylogenetic divergence tree of all labeled SARS-CoV-2 sequences used in this study. Maximum-likelihood tree including all sequences from Northern France, Algerian sequences and publicly available global SARS-CoV-2 sequences, corresponding to the collapsed tree shown in Fig. 3 (same ordering as in Fig. 2 and Fig. 3). GISAID clades are indicated next to the corresponding nodes and branches are colored distinctly. Tips indicate strain names, colored in red for sequences from France, and are noted in bold if discussed in this study.

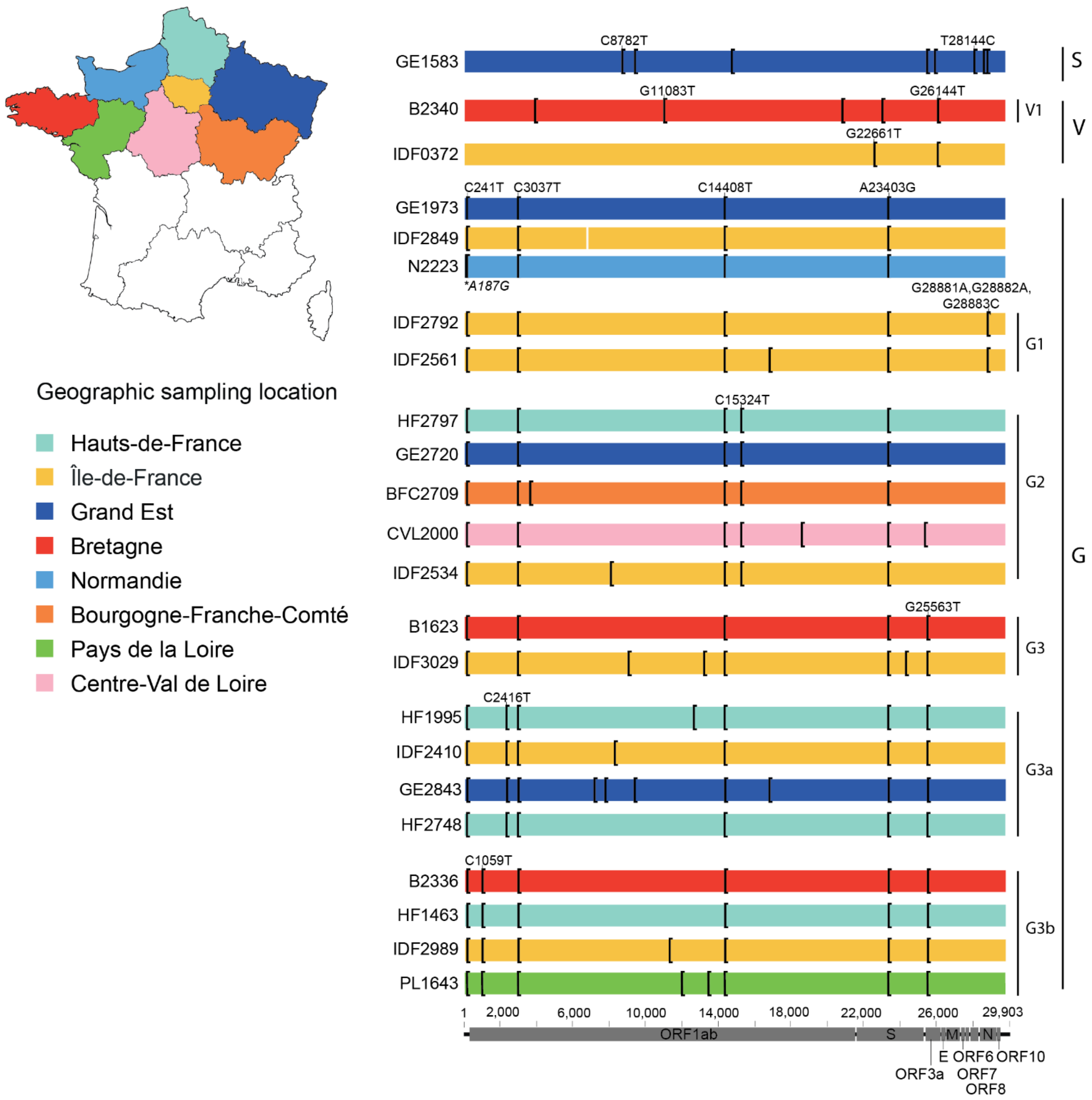
Geographic sampling location
Hauts-de-France
Île-de-France
Grand Est
Bretagne
Normandie
Bourgogne-Franche-Comté
Pays de la Loire
Centre-Val de Loire
GE1583
C8782T
T28144C
S
B2340
G11083T
G26144T
V1
V
IDF0372
G22661T
C241T
C3037T
C14408T
A23403G
GE1973
IDF2849
N2223
*A187G
G28881A,G28882A,
G28883C
IDF2792
IDF2561
G1
C15324T
HF2797
GE2720
BFC2709
CVL2000
IDF2534
G2
G
G25563T
B1623
IDF3029
G3
C2416T
HF1995
IDF2410
GE2843
HF2748
G3a
C1059T
B2336
HF1463
IDF2989
PL1643
G3b
1
2,000
6,000
10,000
14,000
18,000
22,000
26,000
29,903
ORF1ab
S
M
N
E
ORF6
ORF10
ORF3a
ORF7
ORF8

Fig. S2. Single-nucleotide polymorphisms representing the diversity among sequences across the regions of Northern France. Multiple sequence alignment of all SARS-CoV-2 genomes sampled across the northern part of France from different clades and lineages. Single nucleotide variants with respect to the reference (MN908947) are shown as black vertical bars and shared substitutions among the sequences of each clade or lineage are annotated. A substitution only found in sequences from Normandie is noted in italic.

Data S1. (Separate file)

A single Excel document with multiple sheets, representing tables below.

Table S1. Clade defining SNPs according to GISAID nomenclature.

Table S2. Metadata associated with the sequences used in this study.

Table S3. Viral RNA load and genome recovery data.

Table S4. List of collaborators in the RENAL network in the north of France.

References:

1. Quick J, Grubaugh ND, Pullan ST, Claro IM, Smith AD, Gangavarapu K, et al. Multiplex PCR method for MinION and Illumina sequencing of Zika and other virus genomes directly from clinical samples. Nat Protoc. 2017;12(6):1261-76.

2. Kentaro I, Tsuyoshi S, Masanori H, Rina T, Makoto K. Aproposal ofan alternative primer fortheARTIC Network's multiplex PCRto improve coverageof SARS-CoV-2 genomesequencing. bioRxiv 2020.

3. Bolger AM, Lohse M, Usadel B. Trimmomatic: a flexible trimmer for Illumina sequence data. Bioinformatics. 2014;30(15):2114-20.

4. WysokerA F, RuanJ H, MarthG A. DurbinR. 2009a. The sequence alignment/map format and SAMtools. Bioinformatics.25(16):2078-9.

5. Fauver JR, Petrone ME, Hodcroft EB, Shioda K, Ehrlich HY, Watts AG, et al. Coast-to-Coast Spread of SARS-CoV-2 during the Early Epidemic in the United States. Cell. 2020;181(5):990-6 e5.

6. Deng X, Gu W, Federman S, du Plessis L, Pybus OG, Faria N, et al. Genomic surveillance reveals multiple introductions of SARS-CoV-2 into Northern California. Science. 2020.

7. Hadfield J, Megill C, Bell SM, Huddleston J, Potter B, Callender C, et al. Nextstrain: real-time tracking of pathogen evolution. Bioinformatics. 2018;34(23):4121-3.

8. Martin DP, Murrell B, Golden M, Khoosal A, Muhire B. RDP4: Detection and analysis of recombination patterns in virus genomes. Virus evolution. 2015;1(1).

9. Rambaut A, Lam TT, Max Carvalho L, Pybus OG. Exploring the temporal structure of heterochronous sequences using TempEst (formerly Path-O-Gen). Virus evolution. 2016;2(1):vew007.

10. Sagulenko P, Puller V, Neher RA. TreeTime: Maximum-likelihood phylodynamic analysis. Virus evolution. 2018;4(1):vex042.

11. Rambaut A. Phylogenetic analysis of nCoV-2019 genomes http://virological.org/t/phylodynamic-analysis-176-genomes-6-mar-2020/3562020
